# Leisure time activities in adolescents predict problematic technology use

**DOI:** 10.1007/s00787-023-02152-5

**Published:** 2023-02-15

**Authors:** Izaskun Ibabe, Aranzazu Albertos, Cristina Lopez-del Burgo

**Affiliations:** 1 Department of Clinical and Health Psychology and Research Methodology, University College of Psychology, University of the Basque Country UPV/EHU, Avda. Tolosa 70, 20018 Donostia-San Sebastián, Spain; 2 University of Navarra, Pamplona, Spain

**Keywords:** Problematic technology use, Executive functions, Leisure activities, Unstructured leisure, Inhibitory control, Goal setting, Adolescents

## Abstract

The problematic use of technology of children and adolescents is becoming a growing problem. Research has shown that excessive technology use predicts a variety of psychological and physical health problems. The aim of this study was to analyze the role of leisure time activities (structured and unstructured) in adolescents as a predictor of problematic technology use. Participants were 7723 adolescents, of which 55% were girls, from four Spanish-speaking countries (Chile, Spain, Mexico, and Peru) between the ages of 13 and 18 years. The evaluation instrument applied was the YOURLIFE project self-report questionnaire. Two executive functions were measured: goal setting and inhibitory control. Using structural equation modeling, findings indicated that structured leisure time activities predicted less PTU, whereas unstructured activities predicted more PTU, ML*χ*^2^ (69, *N* = 7723) = 806.60; CFI = 0.929, RMSEA = 0.042, and the model had good predictive capacity for PTU (*R*^2^ = 0.46). Structured and unstructured activities also showed indirect effects on PTU through executive functions. As adolescents spent more time in unstructured leisure activities, poorer goal setting, inhibitory control skills, and more PTU were found. The opposite was true for structured leisure time activities. Implications of structured leisure activities to develop executive functioning and to prevent PTU for adolescents are discussed.

## Introduction

Technology has emerged in young people's lives in a rapid and progressive manner in both basic communicative and recreational-expressive roles. Some adolescents and young adults have difficulties in regulating technology use. Problematic technology use (PTU) is characterized by maladaptive behaviors, without reaching levels of addiction [1], and entails negative consequences at a physical, emotional, social and functional levels [2].

A recent study conducted with young people showed three different groups of conflict regarding mobile phone use and inappropriate emotional and communication patterns [3]: a first group (65.9%), characterized by low levels of conflict related to mobile phone abuse and low levels of communicative and emotional use (referred to as non-problematic use); a second group (25.8%), with moderate levels of conflict related to mobile phone abuse and communicative and emotional use (referred to as moderate problematic use); and a third group (8.3%), with high levels in both factors. The negative consequences for young people who use devices, according to an analysis of data from 23,860 Spanish households, are higher risk of mental health problems, significantly reduced sleep hours and greater likelihood of suffering from physical health problems such as obesity [4].

## Executive functions: goal setting and inhibitory control

A variable that may be fundamental in the development of PTU is executive functions (EFs). EFs are all the skills that enable people to have goals and to achieve them through planning and the control of thoughts, emotions and behaviors that interfere with their achievement [5]. In general, EFs play an important role in adolescent development [6] and in childhood predict important life outcomes [7], such as academic and personal development. Goal setting and inhibitory control are two important components of EFs. Goal setting implies organizing a series of steps or a plan toward goal achievement [8], while inhibitory control is the ability to inhibit goal-irrelevant stimuli and create responses using attention and reasoning [9].

There is evidence on the relationship between goal setting and the promotion of autonomy support in physical education lessons, producing positive effects on leisure-time physical activity-related cognition [10]. According to Pichardo et al. [11], emotional control is one the variables with the most direct effects on PTU*.* Some studies found that low levels of emotional regulation or impulsive behaviors were related to PTU [12], or to intensive mobile phone use [13, 14]. Similarly, a recent study based on 3831 school-going adolescents aged 13–18 years showed that inhibitory control as well as goal setting inversely predicted PTU [15].

Inhibitory control is at the core of the emotional regulatory process, and this skill develops meaningfully during the preschool stage [16]. Self-regulation has received massive attention in the last years as a key predictor of a variety of outcomes [17]. It was found that childhood self-regulation is associated with more educational success, physical long-term health and fewer criminal offence outcomes [18]. Although many studies have established a relationship between the presence of a deficit in inhibitory control and PTU, most of them refer exclusively to addiction to online gaming (see the systematic review of Brotons [19]). In terms of communication, adolescents with low self-control are more likely to reply immediately to notifications they receive because they desire immediate gratification and often fail to recognize negative consequences [20]. It seems that loss of control is a fundamental antecedent factor in understanding PTU [21] or dysfunctional smartphone use [22].

## Structured and unstructured leisure time activities

Structured leisure time is defined as any time outside of formal schooling spent in activities organized and supervised by adults [7]. These activities have a challenging component and require concentration [23], provide personal well-being [24], and protect against behavior problems [25]. They also help to exercise self-control, and promote positive psychological adjustment [26]. Astuto and Ruck [27] found significant relationships between artistic activities, sports, and the number of hours young people will invest in extracurricular activities and EFs in childhood. These authors noted that the directionality of links between children’s engagement in play and executive function should be examined in future studies. Specifically, it could be explored whether structured leisure time activity in adolescence improves executive functions and prevents PTU.

In some previous studies, significant associations between engagement in structured leisure activities and academic performance have been found [28], but other studies suggest that participation in structured activities predicts poorer self-directed executive functions [7]. A previous study conducted by Albertos and Ibabe [15] based on adolescents from four Spanish-speaking countries found that two types of structured leisure activities in particular (family activities and recreational reading) were inverse predictors of PTU. In turn, it was found that the participation in structured leisure activities was associated with the existence of family screen time rules [29].

Unstructured leisure activities are characterized by being adult-unsupervised, lacking skill-building aims, taking place in public spaces and having a socializing character [30]. Previous studies in youth centers have revealed that young people’s participation in unstructured activities was related to poor adolescent adjustment and delinquency [31, 32]. To date, some unstructured leisure activities (spending time in youth venues, leisure centers or nightclubs) have been associated with PTU [15]. 

Although the literature is suggestive of factors that may contribute to PTU, there are no previous similar studies analyzing different structured leisure activities. Some previous studies related structured leisure activity to goal setting focused on only one activity, such as physical education [10, 33]. Thus, the primary aim of the present study was to fill this gap in the literature.

## Objectives and hypotheses

The first objective of the study was to analyze problematic technology use and leisure time activities (structured and unstructured leisure) of adolescents for significant differences among four Spanish-speaking countries of origin (Chile, Spain, Mexico, Peru), controlling for age and gender of adolescents. Relevant differences in PTU between countries were not expected, because the results of previous studies have not shown such variation [15].

The second objective was to investigate the explanatory factors of PTU, examining variables associated with leisure time activities and executive functions. A predictive model of PTU based on structured leisure activities, unstructured leisure activities, inhibitory control and goal setting was explored, using structural equation models (SEM). Although a recent study [15] suggested possible relationships between PTU and leisure activities, the dynamic relationships of structured and unstructured leisure activities with executive functions are yet to be discovered. Three hypotheses were posited in relation to this objective:

(a) A higher level of unstructured leisure activities was expected to be associated with more PTU, taking into account the results of a similar study [15] and numerous other studies which have found that unstructured leisure activities could be a risk factor for alcohol and substance use [32] or for delinquent behavior in general [31].

(b) The amount of time adolescents spent in structured activities would relate to their executive functions such as goal setting, because in previous studies, physical education improvement was related to goal setting [10, 33].

(c) Inhibitory control would be associated with a lower level of PTU. Deficits in emotional control have been linked to PTU [11, 12] as well as to intensive mobile phone use [13, 14]. Moreover, it is known that self-regulation has been found to be a predictor of adaptive development across many domains [18].

## Method

### Participants

Sample selection was carried out using the convenience sampling method. The sample consisted of 7723 in-school adolescents between the ages of 13 and 18 years at the time of recruitment (Median = 15, SD = 1.48), of which 55% were girls, from four countries (Chile = 15%, Spain = 24%, Mexico = 29%, Peru = 32%). Participants were from mixed schools (50%) and single-sex schools (50%). Regarding their academic performance, 42% of participants had obtained good grades, 42% had passed all subjects, and 16% had failed one or more subjects. Over 98% lived in an urban area while 2% lived in a rural area. Socio-economic levels may be quite high because the percentage of parents (at least one of the two parents) with university studies was high (Chile 39%, Spain 81%, Mexico 74%, and Peru 69%).

### Instrument and variables

The evaluation instrument used was the YOURLIFE project self-report questionnaire [34], with three different versions depending on age (13, 15 and 17 years). This instrument has been used in several national and international surveys carried out among adolescents and based on other questionnaires [35] (see Appendix).

#### Socio-demographic variables

Information concerning the socio-demographic data of the participants was collected, among them sex, age, country of residence, or parents’ educational level.

#### Problematic technology use

This was assessed on the basis of four questions that included time spent using mobile devices, interacting with peers through social networks, writing emails, chats or tweets, and eating while looking at one’s smartphone (e.g., I spend my time distractedly, looking at my smartphone, tablet or computer, even when I could be doing more productive things). The response format was a Likert-type scale from 0 (Strongly disagree) to 4 (Completely agree). Principal component analysis was performed on four items, one factor based on the standard eigenvalues > 1 criterion was found, explaining 51% of the total variance. The internal consistency of this scale (*α* = 0.68) was acceptable.

#### Structured leisure activities

The frequency of structured leisure activities (volunteer, artistic, sports and family activities) during the last year was measured. The response format had five response options (Never; Less than 1 day a month; 1–3 days a month; 1–2 days a week; and 3 or more days a week).

#### Unstructured leisure activities

The frequency of unstructured leisure activities (in public spaces, leisure centers, and youth venues or nightclubs) during the last year was measured based on four questions, with the same response format as for structured leisure activities.

#### Inhibitory control

This executive function was measured on the basis of responses to one statement (I do things without thinking about them) with five options (Never; Almost never; Sometimes; Almost always; Always). In order to measure inhibitory control, an inverse item was used and scores were inversed.

#### Goal setting

Two executive functions (planning, and achievement of goals) were measured on the basis of responses to two statements (I plan the things I do; I usually finish what I start) with five options (Never; Almost never; Sometimes; Almost always; Always). Principal component analysis was performed on two items; one factor based had eigenvalues > 1, which explained 69% of the total variance. The internal consistency of this instrument (*α* = 0.55) was weak.

### Procedure

Educational centers from four countries were invited to take part by email which provided the link to the website designed to offer detailed information to the participants (http://www.proyectoyourlife.com/). Schools agreeing to participate in the project received a protocol with instructions on the survey process, and on the specified date, each school administered the questionnaire in person during school time. The general design of the study was approved by the Ethics Committee of the University of Navarra, and each new participant school was asked to follow the project’s specific ethical guidelines. The respective ethics committee of each participating country had access to the questionnaire prior to application.

The rationale of the study was explained verbally to the students in all schools involved in the study. Moreover, participants received written information detailing the objectives of the project as well as their rights. The framework questionnaire was administered after parental permission for this research was received. A self-administered anonymous questionnaire was administered. No incentive for participation was offered, but each school was sent a report with the overall results of their center, and the implementation of specific educational programs was encouraged to prevent the problems detected in the study. The data of the present study were collected before the COVID-19 pandemic, and the detailed data collection procedure can be found in a previous publication [34].

### Data analysis

The prevalence rate of PTU was calculated on the basis of participants scoring 4 (Totally agree) on any of the four questionnaire items. Statistical control of potential confounding variables was applied across the whole set of ANCOVA analyses, including as covariates adolescent age and adolescent gender. First, one-way ANCOVA with Country of origin (Spain, Chile, Mexico, Peru) was conducted for PTU. Then, a mixed model ANCOVA 4 (Country of origin) × 4 (Structured leisure: volunteer, artistic, sports, family) was conducted, with Structured leisure as a repeated measures factor. A similar mixed model was executed, with Unstructured leisure (public spaces, nightclubs, leisure centers, youth venues) as a repeated measures factor. In these analyses the Greenhouse–Geisser degrees of freedom correction was applied when necessary. As an index of effect size, the partial eta-squared statistic was used (small effect *η*^2^ = 0.01; medium effect *η*^2^ = 0.06; large effect *η*^2^ = 0.14) [36]. For the posterior contrast analysis, the Bonferroni correction method was used to control the type I error rate, with *α* < 0.05. The correlation matrix between PTU and the study variables (inhibitory control, planning goals and leisure activities) was conducted. These data analyses were performed using IBM SPSS Statistics 27.

Robust statistics are more appropriate when the data are not multivariate normal (Mardia’s normalized coefficient exceeded 23.76). Goodness-of-fit of the model was assessed with the normal theory maximum-likelihood (ML). A number of fit indices were calculated, including: (a) the overall *χ*^2^, (b) Satorra and Bentler [37] (1994), robust maximum-likelihood (S–B), (c) the comparative fit index (CFI), (d) the Satorra–Bentler robust comparative fit index (RCFI), (e) the root mean square error of approximation (RMSEA). The most commonly used criterion for an acceptable fit is CFI ≥ 0.90, and RMSEA ≤ 0.06 [38]. A confirmatory factor analysis (CFA) assessed the adequacy of the hypothesized measurement model and the associations among the latent variables: Problematic technology use (indicators: four items), Goal setting (indicators: planning and achievement of goals), Structured leisure activities (indicators: volunteer, sports, family), and Unstructured leisure activities (indicators: public spaces, shopping centers, youth venues, and nightclubs). The artistic activities indicator was deleted for Structured leisure activities because the factor loading was smaller than 0.32 in an initial CFA. Inhibitory control was included as an observed variable.

In the structural model, Structured leisure activities, Unstructured leisure activities were predictors of PTU. Moreover, Structured and Unstructured leisure activities predicted Goal setting and Inhibitory control, which served as the intervening variables in the relationship between leisure activities and PTU. Significant correlations were allowed among the Unstructured and Structured leisure activities. The estimation of indirect effects was accomplished using a SEM model. These analyses were performed using the EQS 6.2 Structural Equation Program.

## Results

### PTU and leisure activities as a function of country

PTU prevalence was 22% (boys 19%; girls 24%), *χ*^2^ = 32.81, *p* < 0.001, *r* = 0.06. ANCOVA analysis for PTU showed main effects of Country *F*(3, 6821) = 23.50, *p* < 0.001, *η*^2^ = 0.01. According post hoc analysis, Spanish participants (*M* = 1.38) showed less PTU than the other participants (Peruvian *M* = 1.51; Chilean *M* = 1.53; Mexican *M* = 1.59).

The second ANCOVA indicated that Structured leisure, *F*(3, 20,508) = 209.85, *p* < 0.001, *η*^2^ = 0.030, and Country of origin, *F*(3, 6871) = 38.22, *p* < 0.001, *η*^2^ = 0.016, were significant. Moreover, the interaction was significant, *F*(9, 20,508) = 21.24, *p* < 0.001, *η*^2^ = 0.009. Bonferroni post hoc analysis of the interaction indicated that Mexican (*M* = 2.92) and Spanish (*M* = 2.84) participants had higher scores in sports than Chileans (*M* = 2.63) or Peruvians (*M* = 2.52), but in volunteer and family activities, Mexican participants (*M* = 0.96; *M* = 1.93) showed slightly higher scores than the participants of other countries (Chileans *M* = 0.60 vs. *M* = 1.71; Peruvians *M* = 0.67 vs. *M* = 1.77; Spanish *M* = 0.73 vs. *M* = 1.78) (*p* < 0.05).

In the third ANCOVA, results indicated that Unstructured leisure, *F*(3, 20,610) = 290.94, *p* < 0.001, *η*^2^ = 0.041, Country of origin, *F*(3, 6870) = 24.80, *p* < 0.001, *η*^2^ = 0.011, and interaction, *F*(9, 20,610) = 65.02, *p* < 0.001, *η*^2^ = 0.028 were significant. Bonferroni post hoc analysis of interaction indicated that Spanish participants presented more activities in public space (*M* = 3.03) than other participants did (Chilean *M* = 2.50; Peruvian *M* = 2.45; Mexican *M* = 2.40). Moreover, Mexicans showed more leisure center activities (*M* = 1.90) and youth venues (*M* = 1.94) than participants of other countries (Chilean *M* = 1.78 *vs*. *M* = 1.63; Peruvian *M* = 1.61 *vs*. *M* = 1.72; Spanish *M* = 1.56 *vs*. *M* = 1.71) (*p* < 0.05).

### Correlation matrix among observed variables

Table 1 presents the correlation matrix between PTU and the other observed variables. The most notable results were found in the relationship between PTU and unstructured leisure activities (leisure centers *r* = 0.229, *p* < 0.001; nightclubs *r* = 0.206, *p* < 0.001; public space *r* = 0.185, *p* < 0.001), as well as in goal setting (goal achievement *r* = − 0.176, *p* < 0.001; planning *r* = − 0.129, *p* < 0.001). At same time, PTU was associated with less structured leisure (artistic activities *r* = − 0.114, *p* < 0.001; sports *r* = – 0.088, *p* < 0.001).

**Table 1:** Correlation matrix between PTU and the study variables

Variables	1	2	3	4	5	6	7	8	9	10	11
1. PTU	–										
2. Inhibitory control	– 0.243**	–									
Goal setting											
3. Planning	– 0.129**	0.072**	–								
4. Goal achievement	– 0.176**	0.072**	0.388**	–							
Structured leisure											
5. Sport	– 0.088**	0.037**	0.109**	0.154**	–						
6. Volunteering	– 0.035**	0.017	0.094**	0.084**	0.163**	–					
7. Artistic activities	– 0.114**	0.039**	0.111**	0.068**	0.115**	0.261**	–				
8. Family activities	– 0.056**	0.042**	0.141**	0.159**	0.282**	0.157**	0.169**	–			
Unstructured leisure											
9. Public spaces	0.185**	– 0.056**	0.041**	0.050**	0.283**	0.103**	0.085**	0.159**	–		
10. Nightclubs	0.206**	– 0.118**	0.015	0.014	0.112**	0.194**	0.023	0.043**	0.153**	–	
11. Leisure centers	0.229**	– 0.086**	0.020	0.011	0.177**	0.100**	0.082**	0.198**	0.279**	0.236**	–
12. Youth venues^a^	0.083**	– 0.140**	0.016	0.001	0.150**	0.147**	0.036**	0.116**	0.255**	0.348**	0.321**

**: *p* < 0.01; *: *p* < 0.05

^a^Youth clubs and/or friends' houses

Executive functions were related to leisure activities. Planning was mostly related to structured leisure activities (family activities *r* = 0.141, *p* < 0.001; artistic activities *r* = 0.111, *p* < 0.001; sports *r* = 0.109, *p* < 0.001; volunteering *r* = 0.094, *p* < 0.001). In the same way, goal achievement was linked to structured leisure activities (family activities *r* = 0.159, *p* < 0.001; sports *r* = 0.154, *p* < 0.001). Nevertheless, inhibitory control was associated inversely with unstructured leisure activities (youth venues *r* = – 0.140, *p* < 0.001; nightclubs *r* = – 0.118, *p* < 0.001; leisure centers *r* = – 0.086, *p* < 0.001).

### Structural equation modeling

In the confirmatory factor analysis, all factor loadings and latent factors were significant (*p* < 0.001). Fit indices for the CFA model which required no model modification were all acceptable ML*χ*^2^ (68, *N* = 7723) = 799.13; CFI = 0.929; NNI = 0.905; IFI = 0.929; RMSEA = 0.042 [90% CI 0.039—0.045]; S–B *χ*^2^ (68, *N* = 7723) = 735.09; CFI = 0.927; NNI = 0.902; IFI = 0.927; RMSEA = 0.041 [90% CI 0.038—0.043].

**Fig. 1:**
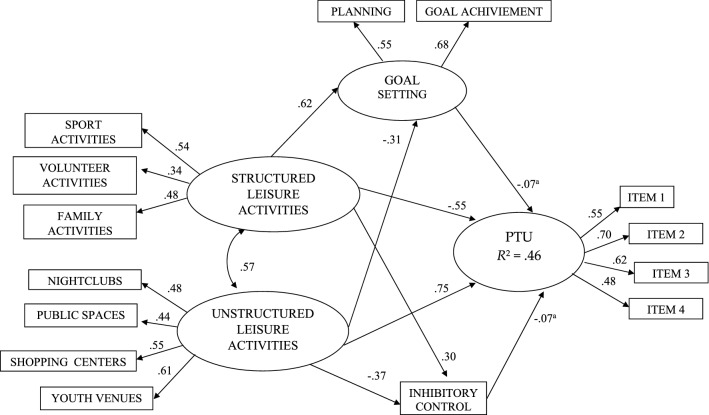
Structural model predicting PTU based on 7723 participants from a community population. All estimated parameters are standardized. All factor loadings, correlation and regression coefficients are significant at *p* < 0.001 except ª*p* < 0.01

The structural model is presented in Fig. 1. This model had good fit statistics: ML*χ*^2^ (69, *N* = 7723) = 806.60, *p* < 0.001; CFI = 0.929; NNFI = 0.906; IFI = 0.929; RMSEA = 0.042 [90% CI 0.039–0.044], S–B *χ*^2^ (69, *N* = 7723) = 752.54, *p* < 0.001; CFI = 0.927; NNFI = 0.904; IFI = 0.927; RMSEA = 0.040 [90% CI 0.038–0.043]. Forty-six percent of the variance in PTU was explained by the model. A direct effect of Structured leisure activities (*β* =  − 0.55, *p* < 0.001) and of Unstructured leisure activities (*β* = 0.75, *p* < 0.001) on PTU was found. Structured activities was a protective factor, while Unstructured activities was a risk factor for PTU. The two types of leisure were significantly and positively associated (*r* = 0.57, *p* < 0.001). Moreover, Structured leisure activities was a significant predictor for Goal setting (*β* = 0.62, *p* < 0.001) and Inhibitory control (*β* = 0.30, *p* < 0.001). At the same time, Unstructured leisure inversely predicted Goal setting (*β* = – 0.31, *p* < 0.001) and Inhibitory control (*β* = – 0.37, *p* < 0.001).

Moreover, we also examined indirect effects mediated through the intermediate variables related to Goal setting and Inhibitory control. Structured leisure activities had significant negative indirect effects on PTU mediated through Goal setting and Inhibitory control (*β* = – 0.072; *p* < 0.01), while Unstructured leisure activities had indirect effects mediated through Goal setting and Inhibitory control (*β* = 0.076; *p* < 0.01).

## Discussion

Nowadays, children and adolescents live in an environment saturated with screen-based technology [39]. It is known that teenagers are a particularly vulnerable population due to the psychological impact of prolonged use of digital technologies. Our findings represent the first demonstration that time spent in a broad range of structured activities outside of formal schooling predicts more goal setting, inhibitory control competences and less PTU, while more time spent in unstructured activities predicts poorer executive functions.

This study was based on a broad sample from four Spanish-speaking countries, and one objective was to analyze PTU and leisure activities by country of residence, controlling for age and gender of adolescents. No relevant differences were found regarding PTU and leisure activities between countries (*η*^2^ < 0.017), but the interaction between country and unstructured leisure activities was slightly higher (*η*^2^ < 0.028). Spanish adolescents showed more activities in public spaces than adolescents from other countries, while Mexican adolescents presented more leisure center activities and youth venues.

The second objective was to show a predictive model of PTU in adolescents based on leisure activities, goal setting and inhibitory control. As hypothesized, a higher level of unstructured leisure activities was associated with PTU, taking into account the results of a similar study [15]. The correlation matrix and SEM model supported this hypothesis, explaining 46% of the variance in PTU. Caldwell and Smith [31] summarized research relating to leisure and crime among adolescents in four perspectives, with one of these perspectives focusing on activity structure*.* The perspective of activity structure argues that time spent in unsupervised activities is likely to develop deviance, while time spent in supervised activities protects against it.

The results of the present study are in accordance with this perspective because the structured leisure time activities factor was shown to be a protective factor against PTU with direct and indirect effect through EFs. In general, structured leisure activity performance protects against risky behaviors [25]. As was expected, structured leisure activities were associated with higher level of goal setting competences according to SEM model. To our knowledge, there are no previous similar studies analyzing different structured leisure activities, because previous studies relating structured leisure activity to goal setting have focused on only one activity, such as physical education [10, 33]. Moreover, in the present study, the relationships between two types of executive functions and PTU have been deepened. Indirect effects of structured activities on PTU were found, with the executive functions being mediational variables. An inverse relationship between goal setting and PTU was reported.

The hypothesis that inhibitory control would be associated with less PTU was confirmed. In previous studies, PTU has been related to inhibitory control [15, 22, 40], impulsive behaviors, lack of concentration, and deficits in emotional control [13, 14]. These results are in line with a large body of evidence supporting that impulsivity-related traits show associations with alcohol use outcomes in adolescence [41]. In general, difficulties in controlling impulses, performing goal-directed behaviors, and limited access to emotion regulation strategies could be risk factors for PTU [42]. Hot executive functions refer to self-control, used when emotions are present, while cold executive functions are skills used when emotions are not strongly present [43]. In adolescence, it may be that hot and cold executive functions develop unevenly and that hot EFs follow a different, perhaps slower, developmental process than cold EFs. This could explain discrepancies between adolescents' theoretical understanding of the possible negative consequences of their behavior and their real-life choices in emotionally charged situations, such as peer pressure [6].

The main limitation of this study is associated with the cross-sectional design; this means that it is not possible to establish the direction of causality between leisure time activities, executive functions and PTU. Regardless of the results obtained from the SEM model, it is crucial to emphasize that accurate predictions cannot be guaranteed by cross-sectional study [44]. PTU (dependent variable) would have to occur after structured or unstructured leisure activities (independent variables), and this must be ensured in the prediction model. The best research designs to test the meditational effects of executive functions in the relationship between leisure time activities and PTU would be longitudinal or experimental designs. Another limitation of the present study relates to the assessment of PTU and executive function measures, which should fit completely with theoretical understanding. Goal setting competence included two executive functions (planning, and achievement of goals). However, goal setting is one of the most frequently used components of behavioral interventions aimed at health behavior change [45]. Furthermore, it is necessary to indicate that PTU and executive functions should be measured with validated instruments with evidence of their psychometric properties.

The internal consistency coefficients do not reach the desired level (*α* ≥ 0.70), but the use of these measures was justified. The insufficient level of the internal consistency coefficient for goal setting might be due to the small number of scale items (two items). According to Dall'Oglio et al. [46], a Cronbach *α* of 0.50 can be legitimate and acceptable with a short scale (i.e., few items). Participants may under-report the severity or frequency of behavior associated with PTU due to a social desirability bias. Moreover, it seems that the socio-economic level of participants was quite high, taking into account parental education. This means that the results of the present study are only generalizable to the middle to upper classes of Spanish-speaking countries. Attention deficit hyperactivity disorder can affect a person in many ways, with impeded executive function skills. However, in the current study, it was not controlled for a potential confounder variable.

The relevance of this study lies in the originality of the subject matter, the predictive power of some executive functions and leisure activities with respect to PTU, as well as the large sample size. To the best of our knowledge, this is the first study which contributes to explaining PTU among young people based on leisure activities using SEM modeling. Future studies could explore the extent to which face-to-face peer interactions and adult-led activities have been replaced by media time.

## Conclusions and practical implications

The more time adolescents spent in structured leisure activities, the better goal setting and inhibitory control competences were shown to be, and PTU was lower. The opposite was true of unstructured leisure activities, which were associated with poorer goal setting and inhibitory control and more PTU. These relationships were robust, but future studies should investigate them further. Although this study has been carried out in an original way, prediction models resulting from cross-sectional designs can be misleading [44]. It is, therefore, necessary to consider inverse causation in the interpretation of the results of the present study. In fact, PTU could cause more time spent in unstructured leisure activities and less time in structured leisure activities. In the same way, EFs (goal setting and inhibitory control) could propitiate more time in structured leisure activities. Future studies could examine the potential bidirectional relationship between leisure activities and PTU.

In any case, it would be interesting to promote structured leisure for adolescents and avoid or control unstructured leisure activities, applying family screen time rules. At the same time, it would be desirable to specifically train parents in the promotion of an adequate balance between digital technology use and other daily activities, adapting the rules to the development stage of their child. Young people most at risk of negative outcomes are those with a high level of emotional problems or low self-esteem [47]. Nowadays, there are new patterns of socialization and use of free time through technologies, but establishing healthy screen time rules taking into account the age of children seems essential to prevent PTU. There is an urgent need to develop prevention programs directed at children and adolescents of different at-risk populations that should be rigorously evaluated, and data should be published on the effectiveness of these interventions [48]. Interventions at an early age would be necessary to support a healthy adolescent relationship with the technologies.

## Data Availability

The research data associated with this paper is avaible on request from the first author, upon reasonable request.
